# An improved machine learning pipeline for urinary volatiles disease detection: Diagnosing diabetes

**DOI:** 10.1371/journal.pone.0204425

**Published:** 2018-09-27

**Authors:** Andrea S. Martinez-Vernon, James A. Covington, Ramesh P. Arasaradnam, Siavash Esfahani, Nicola O’Connell, Ioannis Kyrou, Richard S. Savage

**Affiliations:** 1 Systems Biology Centre, University of Warwick, Coventry, United Kingdom; 2 School of Engineering, University of Warwick, Coventry, United Kingdom; 3 Department of Gastroenterology, University Hospital Coventry and Warwickshire, Coventry, United Kingdom; 4 School of Applied Biological Sciences, University of Coventry, Coventry, United Kingdom; 5 Clinical Sciences Research Institute, University of Warwick, Coventry, United Kingdom; 6 Warwick Medical School, University of Warwick, Coventry, United Kingdom; 7 Aston Medical Research Institute, Aston Medical School, Aston University, Birmingham, United Kingdom; 8 Warwickshire Institute for the Study of Diabetes, Endocrinology and Metabolism (WISDEM), University Hospitals Coventry and Warwickshire NHS Trust, Coventry, United Kingdom; 9 Department of Statistics, University of Warwick, Coventry, United Kingdom; UAE University, UNITED ARAB EMIRATES

## Abstract

**Motivation:**

The measurement of disease biomarkers in easily–obtained bodily fluids has opened the door to a new type of non–invasive medical diagnostics. New technologies are being developed and fine–tuned in order to make this possibility a reality. One such technology is Field Asymmetric Ion Mobility Spectrometry (FAIMS), which allows the measurement of volatile organic compounds (VOCs) in biological samples such as urine. These VOCs are known to contain a range of information on the relevant person’s metabolism and can in principle be used for disease diagnostic purposes. Key to the effective use of such data are well–developed data processing pipelines, which are necessary to extract the most useful data from the complex underlying biological structure.

**Results:**

In this study, we present a new data analysis pipeline for FAIMS data, and demonstrate a number of improvements over previously used methods. We evaluate the effect of a series of candidate operational steps during data processing, such as the use of wavelet transforms, principal component analysis (PCA), and classifier ensembles. We also demonstrate the use of FAIMS data in our pipeline to diagnose diabetes on the basis of a simple urine sample using machine learning classifiers. We present results for data generated from a case-control study of 115 urine samples, collected from 72 type II diabetic patients, with 43 healthy volunteers as negative controls. The resulting pipeline combines the steps that resulted in the best classification model performance. These include the use of a two–dimensional discrete wavelet transform, and the Wilcoxon rank–sum test for feature selection. We are able to achieve a best ROC curve AUC of 0.825 (0.747–0.9, 95% CI) for classification of diabetes vs control. We also note that this result is robust to changes in the data pipeline and different analysis runs, with AUC > 0.80 achieved in a range of cases. This is a substantial improvement in performance over previously used data processing methods in this area. Our ability to make strong statements about FAIMS ability to diagnose diabetes is sadly limited, as we found confounding effects from the demographics when including these data in the pipeline. The demographics alone produced a best AUC of 0.87 (0.795–0.94, 95% CI). While the combination of the demographics and FAIMS data resulted in an improvement on the AUC (0.907; 0.848–0.97, 95% CI), it did not prove to be a significant difference. Nevertheless, the pipeline itself shows a significant improvement in performance over more basic methods which have been used with FAIMS data in the past.

## Introduction

Disease implies a change in a chemical ‘fingerprint’ of the patient’s tissue, either due to a change in the patient’s own metabolism (in cancer or diabetes for instance) or due to the alterations resulting from the pathogens causing the disease (fermentome) [1–5]. In many cases these chemical changes can be detected in natural human waste, be it breath, urine, sweat, stool or other. Many of these chemicals are in the gas–phase, emanating from this biological waste, which is in essence the odour of disease. These odours have been found to be biomarkers for a range of diseases. For instance, acetone, ethanol, methyl nitrate and complex volatile organic compounds (VOCs) have been associated with diabetes mellitus in human breath analysis [6]. Gas phase diagnosis offers considerable potential for the medical profession: it is non–invasive, close to real–time, has minimal consumable cost and has the opportunity to be made point–of–care. For these reasons, researchers have been developing instruments focused on the use of this technology. At first glance, traditional analytical techniques, such as gas chromatography/mass spectrometry (GCMS) would seem the best solution. However, high–end analytical instruments are generally large, bulky, expensive and require specialised infrastructure and trained staff to operate, making them inappropriate for many medical scenarios. Thus efforts have been more targeted towards developing tools that are lower–cost, use air as the carrier gas, are portable and easy to use. The electronic nose is one such instrument. Unfortunately, early instruments suffered greatly from the limited available sensors (providing incomplete chemical information about the sample), sensor drift and poor selectivity making reliable diagnosis challenging. However, since then researchers have been developing increasingly more sophisticated instruments that provide significantly greater chemical information about the sample. Yet this brings its own problem, that of how to analyse the very large datasets generated. Therefore, the use of sophisticated data processing methods are required to improve the effective use of these data.

A recent technological development that falls into this category is Field Asymmetric Ion Mobility Spectrometry (FAIMS). FAIMS can be used to detect (and separate) a complex mixture of chemicals in the gas–phase. Its main use is currently in security applications (e.g. detection of chemical warfare agents), but it is now being applied to more diverse industrial and medical domains. It achieves separation and detection by measuring the mobility of ionised molecules in high–electric fields [1, 7]).

In this study, we adopt a machine learning approach to the analysis of FAIMS data for disease detection. We consider a range of different candidate operational processes, with the goal of defining an improved data processing pipeline. We have chosen an exemplar application, specifically distinguishing between diabetic patients and healthy controls from the chemicals emanating from urine samples.

## Methods

### Pilot study data set

115 urine samples were collected in the University Hospital Coventry & Warwickshire, UK. Of these 115 samples, 72 were from type II diabetes patients and the remaining 43 from healthy volunteers as negative controls. Scientific and ethical approval was obtained from the Warwickshire Research & Development Department and Warwickshire Ethics Committee 09/H1211/38. Written informed consent was obtained from all patients who participated in the study. The demographics of these samples is summarised in Table 1.

**Table 1:** Demographics of the patients from this study.

	Diabetic patients	Volunteers	Overall
Male	43	17	60
Female	29	26	55
Average age	57	45	53
Average alcohol usage	2	5	3
Average BMI (s.d.)	38.3 (10.5)	28.1 (5.96)	34.5 (10.3)
Total	72	43	115

We note that due to the challenges of constructing such a pilot study (our controls are healthy volunteers), there is some degree of demographic mismatch between the disease and control groups. The distributions of age/sex/alcohol use/body mass index (BMI) each overlap between the control and disease groups, but there are statistically significant differences in age and BMI. We can quantify the effect of age/sex/alcohol use/BMI as potential confounding biomarkers, using the ROC curve AUC statistic. To do this we treat each demographic covariate in turn as a single biomarker, which can then be used directly to compute the ROC curve (and hence AUC) due only to that potentially confounding factor. This results in the following AUC scores: Age AUC = 0.73 (0.62–0.83). Gender AUC = 0.6 (0.51–0.69). BMI AUC = 0.79 (0.71–0.87). Alcohol AUC = 0.67 (0.58–0.76). While the BMI result in particular is not ideal (and weakens the claims we are able to make with regards to diabetes diagnosis in this paper), we nevertheless note that the best–performing pipeline scores AUC = 0.85 and therefore offers some evidence that FAIMS data are worth pursuing for diabetes diagnosis. BMI is measured as kg/m^2^ and alcohol usage refers to the number of units of alcohol (measured as 10ml or 8g of pure alcohol) consumed per week.

The samples were frozen at -80°C within two hours of collection for batch sampling. For analysis, samples were thawed to 4°C in a laboratory fridge for 24 hours prior to testing to minimise chemical loss. Analysis was undertaken with a commercial FAIMS instrument (Lonestar, Owlstone, UK).

### Sample testing with FAIMS

Once the samples were thawed, 5 mL of urine sample were aliquoted into a 10 mL glass vial and placed into an ATLAS sampling system (Owlstone, UK), where the sample was heated to 40 ± 0.1°C for 10 minutes. By heating the samples, we assumed we would be sampling the volatile organic compounds (VOCs) present in the samples. Once heated, a flow rate of 500 mL/min of clean, dry air was passed over the sample, mixed with a further 1500 mL/min of clean air and was transferred (through heated transfer lines) to the FAIMS instrument. The flow over of the sample is maintained throughout the measurement phase, so can be considered as dynamic sampling. The ratio of sample to make–up air has been optimised for chemical separation undertaken in previous studies [8, 9]. Once the sample enters the instrument, it is first ionised by a Ni-63 radiation source and is forced through a set of parallel plates. On these plates, an asynchronous electric field is applied, whereby one plate receives a short high value electric pulse, whilst the other a longer, lower value pulse—but with time × pulse height kept constant. Ions will either be attracted to one of the plates (thus drift towards it) or not be affected and exit the plates where it is detected. Ions that touch one of the plates lose their charge and are not detected at the exit. A direct current (DC) compensation voltage is applied to the plates to mitigate the drift of the ions, so that they exit without losing their charge and are detected. Thus by scanning through a range of different electric fields and DC compensation voltages, the instrument is able to measure a range of ions (both positively and negatively charged) with different mobilities. In our case, the compensation voltage used was between +6V and -6V through 512 steps, with the magnitude of the asynchronous electric field stepped in 51 steps. As we measure both the positive and negative ions, this creates 52,214 data points per sample [10, 11].

Each sample was analysed three consecutive times (referred to as a “Run”), with each run taking just over two minutes. This sampling rate offers the best compromise between sample time and sensitivity/chemical information and has been optimised in previous studies [8, 9].

### Workflow

**Fig 1:**
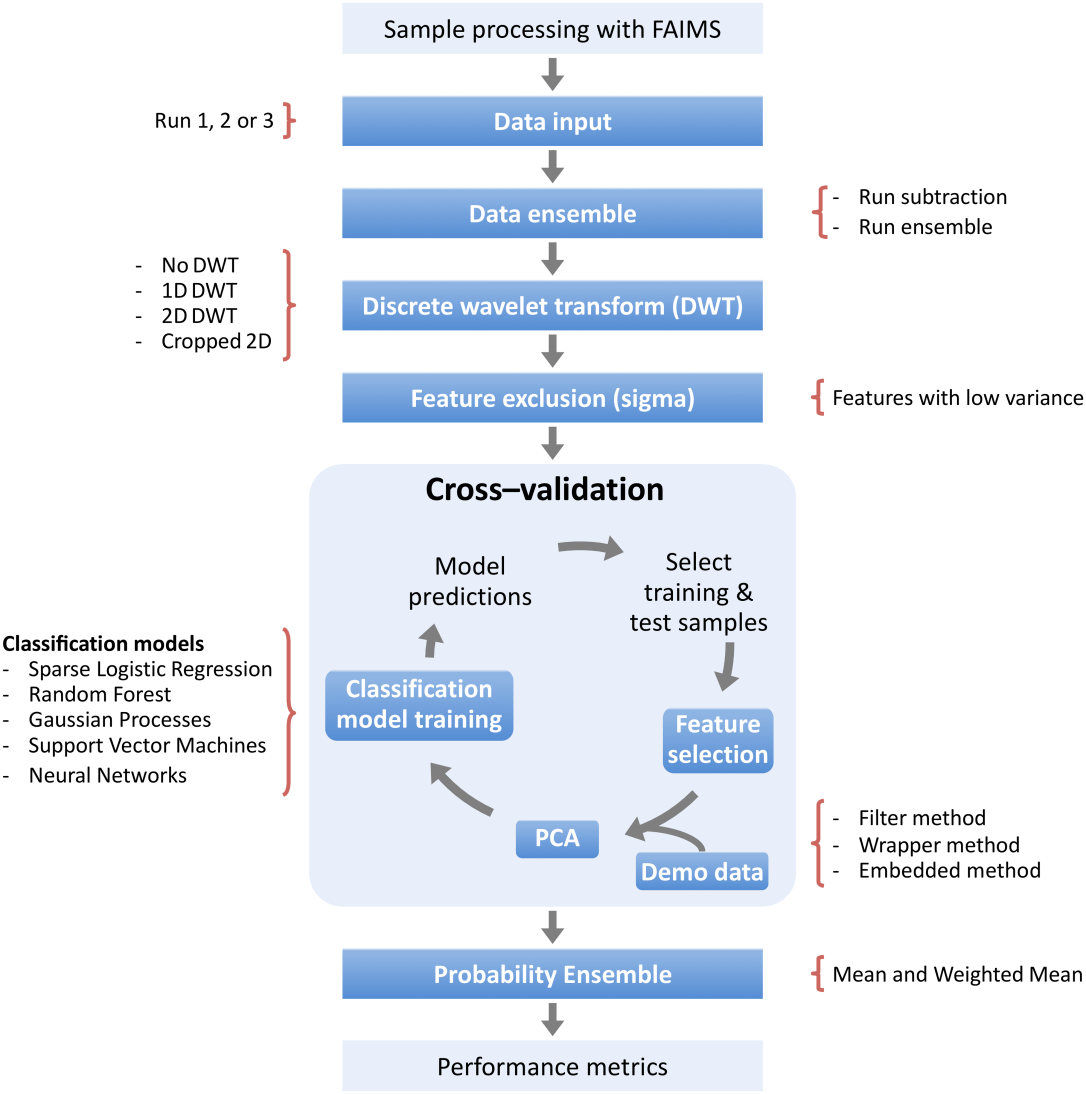
The general workflow of classifying FAIMS data into diseased or non-diseased classes. The steps that were explored are indicated as dark blue boxes. Variations or specification of some steps are displayed at the sides. The order in which the steps and approaches were investigated differs from the order shown in the diagram. Consult the main text for a description of the order. Briefly, the pipeline was compared when using the data of different sample “runs” either individually or in ensembles. Different forms of discrete wavelet transforms (DWT) were considered, as well as a feature exclusion step based on the feature variance. Within the cross–validation cycle, we evaluated three different feature selection methods (filter, wrapper and embedded), as well as a post–filter selection principal component analysis (PCA) step and the inclusion of the demographic data as features. Finally, we also explored ensemble steps at the classifier model probability level. See main text for details and the order in which the pipeline was explored.

The general workflow is summarised in Fig 1 and was developed in R (v 3.0.2). The aim of this study was to explore different steps and approaches to maximise the classification model performance, using diabetes as a medical exemplar. To avoid trying exhaustively every possible combination of pipeline elements, we considered each stage separately, identifying the best–performing option and then keeping that option for all subsequent analyses. As such, this is a greedy search strategy for the optimal pipeline configuration. The steps and approaches are indicated in Fig 1 as filled boxes (dark blue) and we have used their legends (e.g. “data input”) as section headers throughout the paper. The following paragraph outlines the order in which the pipeline was investigated and in which the paper is presented.

**Fig 2:**
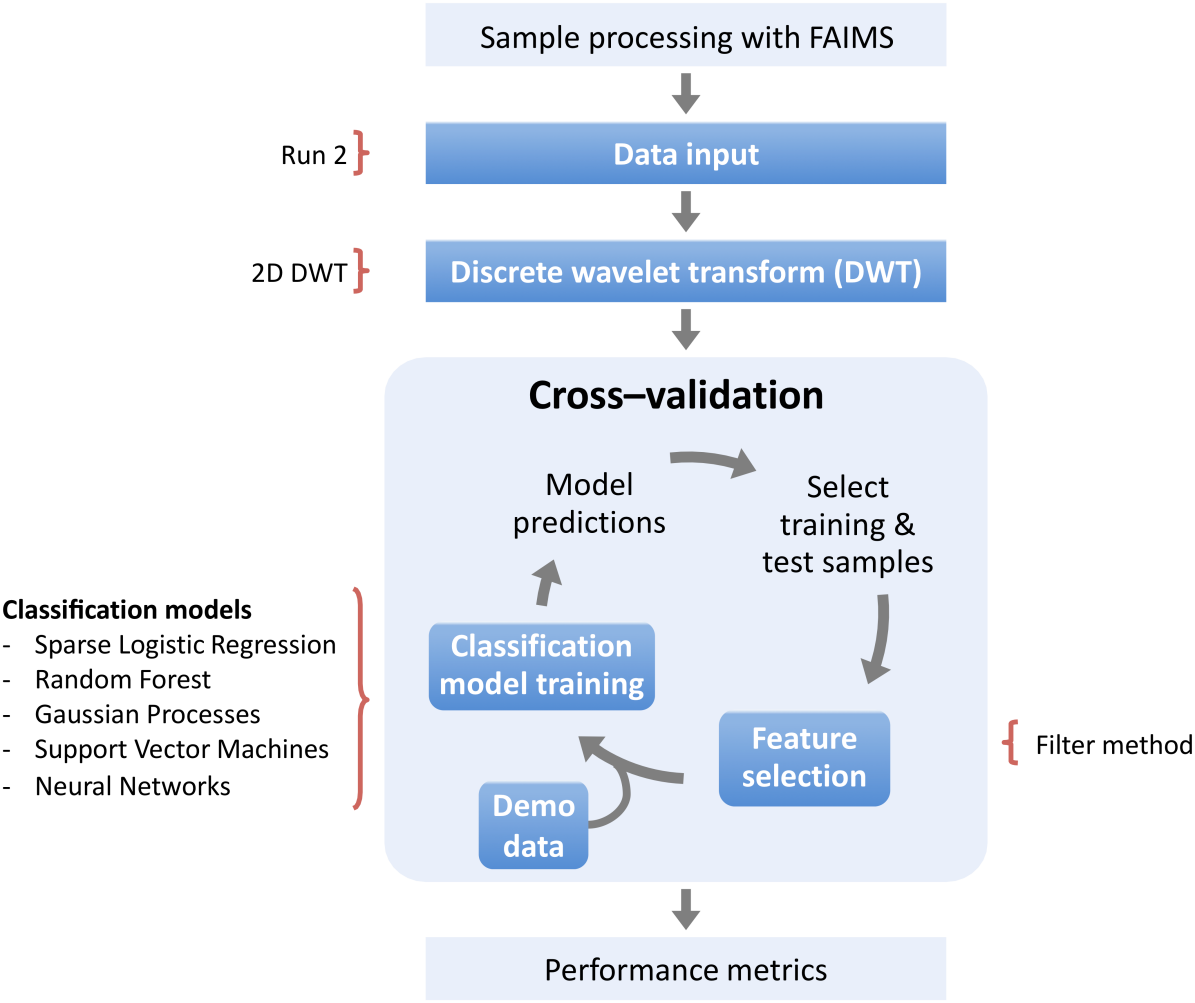
The recommended pipeline for classifying FAIMS data into diseased or non-diseased classes resulting from this study. We found that “run” 2 data with a 2D wavelet transform were the better performing steps prior to the feature selection. The filter method with an *nKeep* parameter value of 2 perform best and with minimal algorithm run time. The addition of the demographic data as features to the wavelet transform FAIMS data resulted in a higher AUC score, although it was not found to be a statistically significant finding. However, these data might prove informative in a larger-scale pilot analysis. Overall, no classifier model was found to out–compete the others and we therefore suggest to use all five, until further research determines a “clear winner”. See main text for details and discussion about our findings.

We first explored the pipeline performance when using the data of different sample “runs”. We next investigated the use of discrete wavelet transforms (DWT) followed by a feature exclusion step based on the feature variance. Afterwards, we examined the feature selection filter method and its parameter *nKeep*, and the use of a principal component analysis (PCA) for an additional feature selection step. We compared our selected feature selection method, the filter method, against two other common methods: wrapper and embedded. We later perform ensembles both at the data input and at the model prediction probability levels. Finally, we explored studied the performance of our pipeline when including demographic data as features together with the wavelet transformed FAIMS data. The following sections describe the methods employed for different steps and approaches, while the *Results and discussion* section describes our findings. These make up our recommended pipeline which is the result of the work presented in this paper, summarised in Fig 2.

#### Data inputting

**Fig 3:**
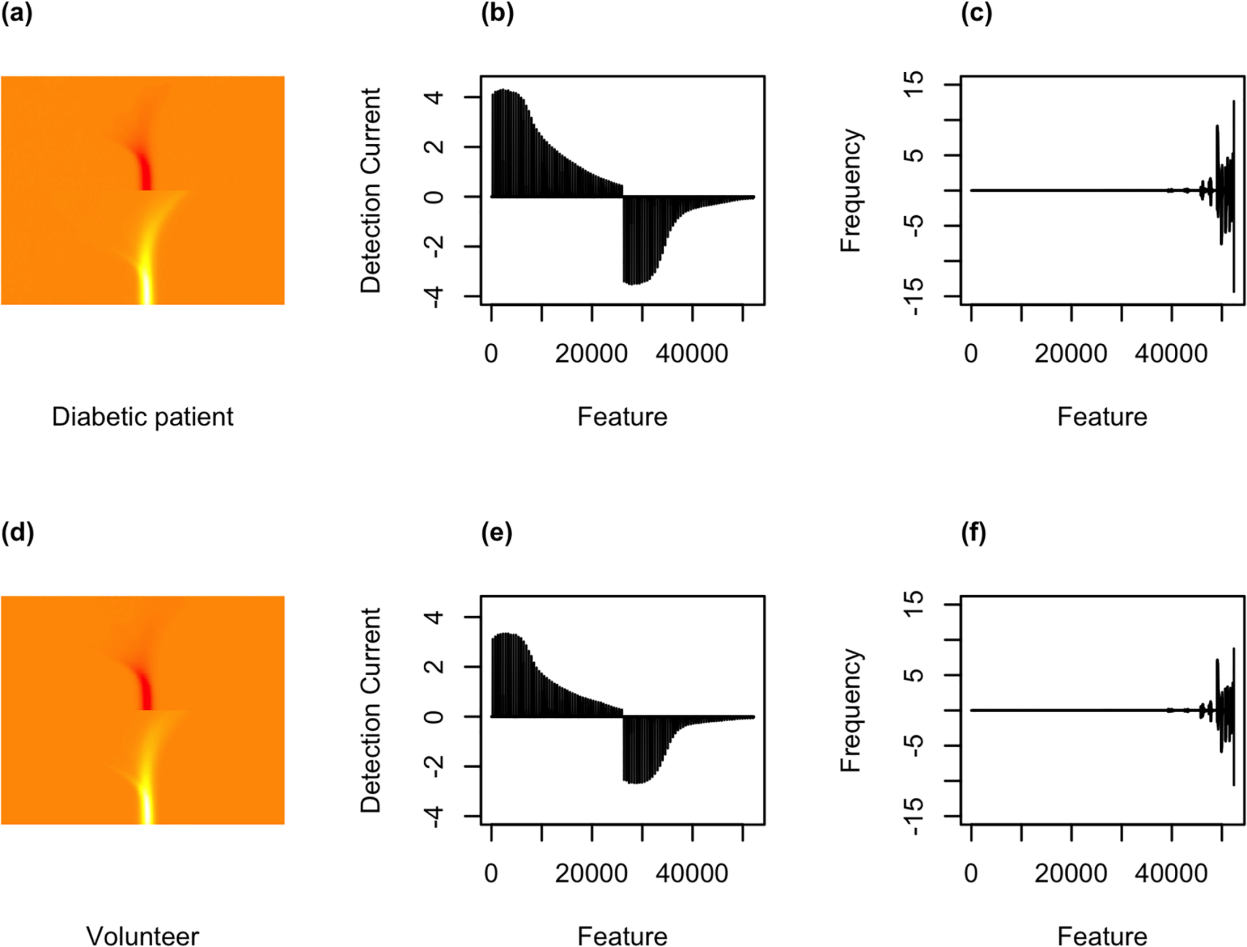
Data visualisation. (a) Heat map of FAIMS data for a diabetic patient. (b) Linearised data without wavelet transform. (c) Data with one–dimensional (1D) discrete wavelet transform (DWT). (d-f) show the equivalent plots for a member of the control group (volunteer).

The current detected of the positive and negative ion values of the desired FAIMS sample “run” (defined in *Sample testing with FAIMS* subsection) was concatenated in a single vector. A data matrix was constructed by including the detected current values as columns or features (52,224 in total) for each sample (rows). A heat map of the data of a diabetic patient can be observed in Fig 3a as an example, as well as its corresponding data plots, either as a detection current vs feature (original data, Fig 3b) or the wavelet coefficients (Fig 3c). The corresponding figures for a volunteer sample can be observed in Fig 3d–3f. The pipeline performance was compared when using the data of different “runs”.

#### Discrete wavelet transform (DWT)

To compress the high dimensional dataset, a discrete wavelet transform (DWT) was applied. This transform aids in extracting subtle chemical signals hidden within a much larger signal. A one–dimensional (1D) DWT has been our default approach in previous work [8, 9], using Daubechies’ ‘least–asymmetric’ wavelets with the *wavethresh* package [12, 13]. While a 1D wavelet transform attempts to compress a signal in its ‘linear’ form, two–dimensional (2D) wavelets apply a 1D wavelet in the x, y and diagonal directions of a matrix. It was assumed that a 2D wavelet transform would be better able to conserve the structure of the data observed in Fig 3a and 3d. Therefore, to perform the 2D wavelet transform, the linearised data was transformed into a matrix. To satisfy the 2D DWT criteria of having a squared 2^*n*^ matrix, zero columns were added to the data. Furthermore, model performance was also studied when the main data structure of the FAIMS data was preserved by ‘cropping’ the 512 x 102 matrix (padded to a 512 x 512 matrix), resulting in a 256 row by 102 column matrix or a 128 row by 102 column matrix. Zero columns were added to obtain a squared matrix as well with 256 x 256 and 128 x 128 matrices, respectively. The 1D, 2D, 2D cropped (256 x 256) and 2D cropped (128 x 128) yielded, respectively, 52,395, 349,525, 87,381 and 21,845 features.

#### Feature exclusion

We expected only a subset of the data features to be informative about the diabetes/control classification task. We therefore used two stages of feature selection to remove the uninformative features, thereby both improving the speed of analysis and also removing noisy features that would degrade the predictive power of our classification algorithms.

The first stage is a simple threshold on the standard deviation of the features across all samples (parameter referred to as *sigma*) before the cross–validation step. This is mainly for practical reasons, as we expected some of the wavelet transformed values to be very uniform. This occurs for two principal reasons. Firstly, the FAIMS system systematically samples two different voltage settings in order to generate the 52,214 features. Some regions of this sampling (e.g. the edge regions) are known to contain negligible signal simply because virtually no ions are observed there. This can be seen, for example, in Fig 3a. Secondly, the wavelet transforms require data of size 2^*n*^ (or 2^*n*^ by 2^*m*^ in 2D), so the transformed vectors/matrices are as standard ‘padded’ with zeros. This is a standard procedure for working with wavelets and does not affect the wavelet coefficients of interest, but it does lead to wavelet–transformed features which have very low (even zero) variance because they relate to this padding. By removing features with very small *sigma*, we remove these uniform features and therefore make the subsequent processing steps faster and less memory–intensive. A range of *sigma* values was explored (see Results and discussion), but unless otherwise stated, this threshold (*sigma*) was set to zero (which would therefore only remove features with zero–variance, which result from zero–padding the data matrix to allow for the wavelet transform).

#### Feature selection: Filter method

The second stage of feature selection aimed to feed the classifiers with the most informative features and was implemented within each cross–validation step (i.e. features are selected only on the basis of the training data subset within a given fold of the cross–validation to make the cross–validation a fair test).

To this purpose, we used a filter method, implemented with a Wilcoxon rank–sum test for each feature in turn. We then kept only the ‘n’ features with the lowest p–values (parameter referred to as *nKeep*). A range of *nKeep* values was explored, but *nKeep* = 2 was used throughout the analyses unless otherwise stated. We note that some of the classifiers we use have built–in feature selection (e.g. Random Forest, Sparse Logistic Regression). In these cases, the filtering–based approach discussed above can be regarded as a fast means of screening out features, in order to speed up the classification algorithms.

#### Principal component analysis (PCA)

To reduce input dimensionality, while maintaining as much of the expected variance as possible due to the nature of the data, principal component analysis (PCA) was implemented with the *princomp* function in the *stats* package [14]. This approach has the additional advantage that it makes no use of the sample target values (diabetic or control). To meet the *princomp* function requirements, the filter method was used to have as many features as training data elements or less (determined by *nKeep*; 103 or 104 depending on the cross–validation iteration). This step returned in average 102 features (range of 99 to 104). The PCA was then carried out with the selected features and the first *n* principal components (required to account for at least 95% of the variance) were used as input for the classification models, always keeping at least the first two for the classification models to work.

#### Feature selection: Method comparison—Wrapper & embedded methods

We further investigated the performance of our filter method approach compared to a wrapper method using all five models and to in–built embedded method of the Sparse Logistic Regression and Random Forest classifiers, in terms of predictive ability and computational time. For the embedded method comparison, we ran those two models individually with the entire feature set and recorded the run time and performance metrics.

We used the *stepAIC* function in the MASS package [15] to implement the wrapper method, as it is a widely used function designed for stepwise feature selection [16]. None of the packages used for the classifier models were compatible with the *stepAIC* function. We therefore used a Generalized Linear Model (implemented with the *glm* function in the *stats* package) to perform the stepwise selection of the features to be used in the classifier model training step. Five random features were chosen to start the stepwise *glm* model fitting allowing for both addition and removal of features (function set to ‘both directions’ with no printed output). The *stepAIC* function returns the model with the features used when the addition or removal of features ceases to minimise the Akaike information criterion (AIC). For this analyses, we used a 2D DWT (uncropped), *sigma* = 10^−7^ and *nKeep* = 2, without PCA.

#### Machine learning algorithms for classification

A 10–fold cross–validation was used. This approach has the advantage of using all available data, while balancing the tradeoff of bias and variance [17, 18]. At each fold, ca. 90% of the data was used as the training set. The classified models produced predictions for the remaining data (test set), allowing predictions for the entire data set to be obtained. The five classification models used are listed below. The machine learning algorithms were implemented with the R packages mentioned below with the default parameters unless otherwise stated (consult package documentation for parameter definitions).

- **Random Forests** (R package *randomForest*; [19, 20])—500 trees to grow while assessing the importance of the predictors. *Non–default parameters*: importance = TRUE
- **Sparse Logistic Regression** (SLR, R package *glmnet*; [21])—binoamial response type with a lasso penalty. *Non–default parameters*: family = “binomial”, alpha = 1
- **Support Vector Machines** (R package *kernlab*; [22])—Radial Basis kernel “Gaussian” with a sigma value of 0.05 with a cost of constraints violation of 5 performing a 3–fold cross validation on the training data to assess the quality of the mode. *Non–default parameters*: kernel = “rbfdot”, prob.model = TRUE, kpar = list(sigma = 0.05),C = 5,cross = 3
- **Artificial Neural Networks** (R package *neuralnet*; [23, 24])—resilient backpropagation with weight backtracking, 1 hidden neurons (vertices) in each layer. *Non–default parameters*: linear.output = FALSE, likelihood = TRUE
- **Gaussian Processes** (R package *kernlab*; [22, 25])—Radial Basis kernel “Gaussian” without scaling and a tolerance of termination criterion of 0.01. *Non–default parameters*: scaled = FALSE, tol = 0.01

#### Performance metrics

We used the R package *pROC* to produce the receiver operating characteristic (ROC) curves for each classification model under the different operational and data processing conditions [26]. The area under the curve (AUC) was used as metric for model performance and for classifier comparison. The AUC’s 95% confidence intervals (CI) were calculated with 2000 stratified bootstrap replicates. The threshold to balance the sensitivity (*SE*) and specificity (*SP*) was found at the point where (*SE* − 1 + *SP*)^2^ + (*SP* − 1 + *SE*)^2^ was maximal across their paired values. The sensitivity and specificity confidence intervals (CIs, 95%) were given by the confidence intervals for the probability of success resulting from a binomial test of the true positives given the total positives and true negative given the total negatives, respectively.

We note some interesting discussions in the literature on the choice of the AUC statistic as a metric for comparing classifiers. [27] shows that if one assumes known optimal classifier thresholds but an unknown misclassification cost ratio, then AUC is incoherent for the comparison of classifiers in the sense that the distribution for the cost ratio is classifier–dependent (so we would not be comparing like with like). This is further examined by [28], who show that this incoherence arises because of the assumption that the optimal classifier thresholds are known. If one instead integrates them out as unknown, this incoherence disappears and AUC remains a reasonable metric to use in classifier comparison. We include this to note that choice of metric is hard, and can have hidden statistical depths that need proper consideration.

#### Ensembles

To attempt to further improve the predictions, we investigated a number of ensembling methods. These methods seek to combine information across the three FAIMS runs. We note that it is important to consider the differences in performance of these three runs, as it relates to the precision of the system/method used, which is highly important in a clinical environment.

- **Run subtraction** The data from Runs 1 and 3 were subtracted from each other to investigate the possible effect of sample degradation on classifier performance.
- **Run ensemble** This was a simple averaging of the data for the three FAIMS runs performed for each urine sample. The goal here was to assess whether repeated measurements could be averaged to improve signal–to–noise, and hence classification results.
- **Probability ensemble** This attempted a similar goal, but by producing predictions separately for each of the three runs, and then averaging the prediction probabilities. The idea here was to account for any difference in structure across the runs, while still asking the same basic question about discriminating between disease and control.

#### Use of demographic data as features

To investigate whether the demographic data are potential confounders, we also ran the machine learning models using only the four demographic variables (sex, age, alcohol use, BMI) as features. A strong result using only these would indicate that the demographic data do indeed confound the VOCs result. We also ran analyses combining the four demographic features with the two Wavelet–VOC features selected by the filter method, to explore whether these features contain complementary information as measured by improvement in predictive ability.

## Results and discussion

The main aim for this study was to develop a data processing pipeline using a machine learning approach as part of a diagnostic tool to distinguish between diabetic and control patient samples. Consequently, we explored how the implementation of different operational steps affected the classification model performance and how the data itself could be better exploited to yield improved results. These steps and approaches are summarised in Fig 1 and the order in which they were investigated are described in the *Methods* section and the results presented below. Based on our findings, we recommend a pipeline to be used for future FAIMS data analysis (Fig 2). For the diabetes data, the overall best classifier was found to be Sparse Logistic Regression (SLR). The full results for all classifiers are presented in the Supporting information; in the main paper, we restrict ourselves to discussion of the SLR results by way of being an exemplar. We note that in our experience, each of these classifiers can be effective on various FAIMS data sets, and we have not seen one clear winner, in terms of a classifier that is uniformly superior across many different FAIMS data sets (data not shown). We therefore recommend all classifier models to be used until a ‘clear winner’ can be found by testing the pipeline with a larger data set.

Example data is shown in Fig 3 as representations of a typical sample analysed from a diabetic patient (a–c) and a volunteer (d–f). Although the heatmaps in Fig 3a and 3d are not visibly different, it can be observed in Fig 3b and 3e that the range of the current detected (y–axis) is larger for the diabetic patient, which is also reflected in the frequencies obtained by the one–dimensional (1D) discrete wavelet transform (DWT), suggesting that both samples resulted in different measurements. This observation supports the association of VOCs with diabetes mellitus [6]. Since the phase of the sample being analysed was the gas phase, this suggests that there could be a difference between the VOCs measured from a diabetic patient’s sample than from a volunteer (control group). Therefore, we expected classifier algorithms to be able to “learn” to distinguish between them.

### Data inputting

Cross–validation runs of these data showed clearly that the ‘Run 2’ data were the best performing for a range of different classifiers and pipelines. Therefore, unless otherwise stated, all results presented in the main body of the paper are for ‘Run 2’ data (see Table 2, S1, S2 and S3 Tables). We also note that the ‘1D DWT’ result in Table 3 (complete information in S4 and S5 Tables) is the default pipeline that was used, prior to the work in this paper. For each subsequent subsection, we update what the ‘default’ pipeline is on the basis of our results. We are therefore improving our data pipeline in a stepwise manner. The complete performance metrics are given in the Supporting information. In the main paper, we show as an exemplar the results for Sparse Logistic Regression (SLR).

**Table 2:** Model performance comparison with the use of different runs.

	Run 1	Run 2	Run 3
AUC	0.739	**0.825**	0.805
–CIs	(0.648–0.83)	(0.747–0.9)	(0.722–0.89)
Sensitivity	0.528	0.625	0.833
–CIs	(0.353–0.593)	(0.264–0.497)	(0.0892–0.273)
Specificity	0.93	0.953	0.674
–CIs	(0.0146–0.191)	(0.00568–0.158)	(0.191–0.485)

Model performance (confident intervals; CIs) are reported for the Sparse Logistic Regression algorithm.

**Table 3:** Model performance comparison of use of raw FAIMS data and wavelet-transformed FAIMS data.

	no DWT	1D DWT
AUC	0.682	**0.814**
–CIs	(0.584–0.78)	(0.736–0.89)
Sensitivity	0.444	0.569
–CIs	(0.434–0.673)	(0.314–0.553)
Specificity	0.907	0.977
–CIs	(0.0259–0.221)	(0.000589–0.123)

Model performance (confident intervals; CIs) are reported for the Sparse Logistic Regression algorithm.

### Discrete wavelet transform (DWT)

We first explored the effect of using the FAIMS data directly or using the coefficients of a one–dimensional discrete wavelet transform as input for the classification models. An example of the FAIMS data and its corresponding wavelet transform for a diabetic patient are shown in Fig 3.

#### Two–dimensional DWT

The classification model performance was explored when using a two–dimensional (2D) DWT. In order to achieve this, the data were transformed into a matrix. To satisfy the DWT criteria of having a squared 2^*n*^ matrix, zeros were added to the data. As the 512 x 512 matrix resulted in a wide space with seemingly no information, the portion of the matrix with structure was ‘cropped’ and the resulting classification performance evaluated as a 128 x 128 matrix. Table 4 shows the results for the SLR performance across the three forms of 2D wavelet transform and the 1D wavelet transform. The 2D wavelet transform showed consistently better predictive performance across all the classifiers (see S6, S7 and S8 Tables for other classifier metrics). We therefore recommend a simple 2D wavelet transform as part of the pipeline.

**Table 4:** Model performance comparison using different of 2D wavelet transforms.

	2D DWT	2D DWT (256 x 256)^a^	2D DWT (128 x 128)^a^
AUC	**0.825**	0.824	0.824
–CIs	(0.747–0.9)	(0.746–0.9)	(0.746–0.9)
Sensitivity	0.625	0.639	0.597
–CIs	(0.264–0.497)	(0.251–0.483)	(0.289–0.525)
Specificity	0.953	0.93	0.977
–CIs	(0.00568–0.158)	(0.0146–0.191)	(0.000589–0.123)

Model performance (confident intervals; CIs) are reported for the Sparse Logistic Regression algorithm.

^*a*^ Cropped matrix dimensions. Baseline matrix dimensions is 512 x 512.

### Feature exclusion

We expected only a subset of features to be informative. Therefore, an approach was to exclude features with a low standard deviation across the samples, since this would indicate that all samples had a similar value for that feature and would not be informative to the classification models. We investigated the effect that feature exclusion would have on model performance by setting a threshold referred to as *sigma*. Features with a standard deviation lower than *sigma* were excluded from further processing. We observed that the models performed similarly at low *sigma* threshold values, after which the performance deteriorated (see S1 Fig). This reflects that the two sample Wilcoxon test used to select the classification model’s input is effective in determining which features contain the most information that could allow the models to better classify the samples into the two categories. An advantage of setting *sigma* > 0 would be to reduce the number of features tested in the feature selection step. However, in practice we found it gave no advantage in predictive ability and only a minor boost in speed of computation, so for the sake of simplicity we recommend that *sigma* be kept at 0 in the pipeline (or that this step is omitted entirely).

### Feature selection: Filter method

**Fig 4:**
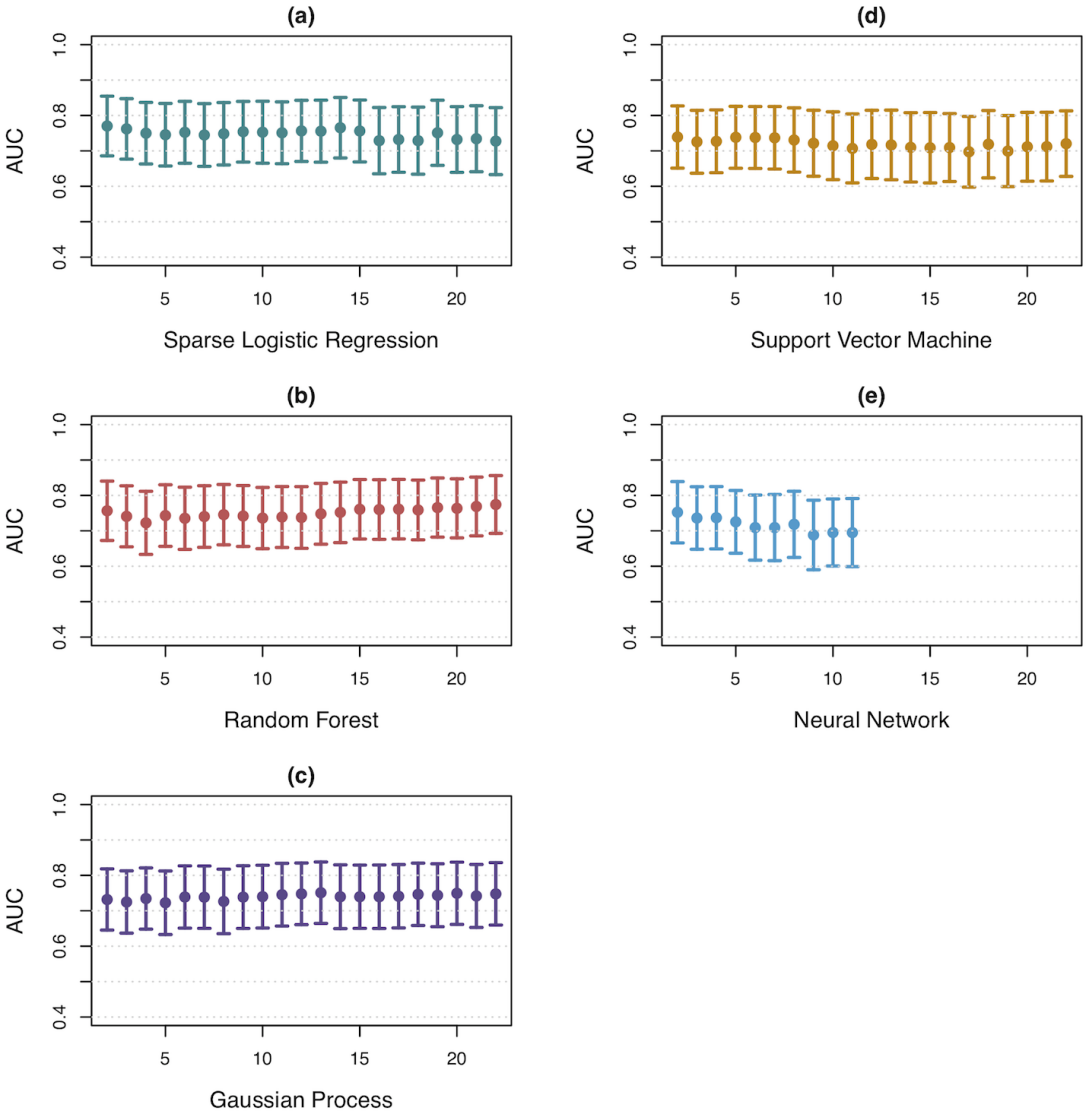
Classification model performance for each model across a range of *nKeep* values. Error bars show the 95% confidence intervals. Neural Network cannot be used with more than 11 features.

An important aspect of any pipeline is defining the parameter values. One was the number of features to be used in the classification models when implementing the filter method. Fig 4 summarises the performance of the different classification models, as measured by the AUC, over an *nKeep* range of 2 to 22. Most models performed stably across the *nKeep* range. The exception was the Neural Network model, as it failed to compute with an *nKeep* > 11. As this model was not performing better than the others, we did not investigate why it might have failed. It is recommended that the pipeline be carried out with *nKeep* = 2, since it results in the least input dimensionality possible. All further work was used with *nKeep* set to 2.

We observed that the same two features were selected in all cross–validations folds, except in one of them where one of the features was different. Due to the data transformations we performed and recommend as part of the pipeline, the features selected do not have a direct physiological meaning. Extensive chemicals analysis could perhaps be used to determine whether specific chemicals were responsible for these informative features, but that is beyond the scope of this paper.

### Principal component analysis (PCA)

Since consecutive detections led to a fingerprint–like signal, an assumption was that the features were correlated due to the nature of the readings. Therefore, principal component analysis (PCA) was implemented as a means of input dimensionality reduction while conserving as much as the variance as possible and exploiting the nature of the correlated features. Our pipeline was set to use as many principal components (PCs) as required to account for at least 95% of the variability of the data (with minimum two PCs as required by the PCA function (see Methods). In all cross-validation folds, however, only two PCs were required to account for our variability threshold. Table 5 and S9 Table compare the baseline run (“run” 2, 2D DWT) against the implementation of PCA also run with a 2D wavelet transform. Again, the SLR model is shown for illustration purposes. PCA degrades the predictive performance, and so we conclude that it is not useful as part of the FAIMS pipeline and recommend this step to be omitted.

**Table 5:** Model performance comparison of PCA implementation.

	no PCA	PCA
AUC	**0.825**	0.8
–CIs	(0.747–0.9)	(0.717–0.88)
Sensitivity	0.625	0.694
–CIs	(0.264–0.497)	(0.202–0.425)
Specificity	0.953	0.814
–CIs	(0.00568–0.158)	(0.0839–0.334)

Model performance (confident intervals; CIs) are reported for the Sparse Logistic Regression algorithm.

### Feature selection: Method comparison—Wrapper & embedded methods

To demonstrate that our default filter method for feature selection performed better both in terms of predictive classifier ability and computation time, we compared it with two commonly used feature selection methods, embedded method and wrapper method. We ran the three feature selection method variants (filter, embedded and wrapper) and recorded the algorithm run time and the performance metrics. A comparison table can be found in the Supporting information (S10 Table). We tested the classifier performance using the embedded method of both Random Forest and Sparse Logistic Regression models. We ran these models individually with the entire feature set. The results for Sparse Logistic Regression (SLR) can be seen in Table 6 (see S10 Table for the Random Forest results). The reduced run time for SLR is due to the fact that all classifier models were trained sequentially when the filter method was implemented, while a single model was trained while using the embedded method. The AUC resulting from this method was lower than that obtained from the filter method for both models, suggesting that filtering does indeed remove uninformative features. We therefore recommend using the filter method over the embedded method.

**Table 6:** Feature selection method comparisons.

	FILTER	EMBEDDED*
n Features	2	87983
AUC	**0.825**	0.824
–CIs	(0.747–0.9)	(0.746–0.9)
Sensitivity	0.625	0.694
–CIs	(0.264–0.497)	(0.202–0.425)
Specificity	0.953	0.884
–CIs	(0.00568–0.158)	(0.0389–0.251)
**Run Time**	8.81 min	54.87 min

* Only SLR model run

Model performance (confident intervals; CIs) are reported for the Sparse Logistic Regression algorithm.

Furthermore, we compared the classifier model performance and run times when a wrapper method was implemented using a stepwise model selection function (see Methods). When we attempted to run this step with all the features, we estimated that the algorithm would require over 25 days to complete. Such overhead would prove impractical for any application. To reduce the run time, but still be able to show the predictive ability, we pre–selected a number of features using the filter method to choose the features with the lowest p–values (or greater variance). Table 7 shows the AUC values obtained when a number of pre–selected features were used and the algorithm run time. It can be observed that none of the AUC obtained were greater or equal to that achieved by the filter method. Furthermore, the use of an increasing number of features in the wrapper method resulted in worse performance, suggesting that the filter method is able to remove uninformative features, allowing for better classifier performance. Additionally, as the number of features increased, the algorithm run time increased exponentially, rendering the use of a large feature set impractical. We therefore recommend using the filter method over the wrapper method as well, using *nKeep* = 2 as determined before.

**Table 7:** Feature selection method comparison.

WRAPPER						
**n Features**	100	250	500	1000	2000	3000
AUC	0.739	0.765	0.751	0.762	0.756	0.703
–CIs	(0.645–0.83)	(0.672–0.86)	(0.652–0.85)	(0.672–0.85)	(0.666–0.85)	(0.603–0.8)
Sensitivity	0.681	0.722	0.764	0.625	0.694	0.764
–CIs	(0.214–0.44)	(0.179–0.396)	(0.144–0.351)	(0.264–0.497)	(0.202–0.425)	(0.144–0.351)
Specificity	0.767	0.767	0.721	0.814	0.744	0.628
–CIs	(0.118–0.386)	(0.118–0.386)	(0.153–0.437)	(0.0839–0.334)	(0.135–0.412)	(0.23–0.533)
**Run Time**	100.34 min	9.64 min	511.39 min	635.98 min	438.41 min	709.06 min

Model performance (confident intervals; CIs) are reported for the Sparse Logistic Regression algorithm.

### Ensembles

Until this point, all data processing had been done with the data from Run 2, which gave the best predictive performance (see Table 2). However, we wished to also explore some options for combining multiple runs and/or classifiers, to improve prediction. As FAIMS analysis uses both high continuous flow rates and has a long analysis time, sample degradation can occur over the three runs, with both low and high molecular weight molecules being sampled at the first run and possibly just the higher molecular weight molecules in the last run (due to sample degradation). By subtracting Runs 1 and 3, it is possible to remove the background of larger molecules to allow investigation of these smaller molecules to investigate if they have any clinical utility. As can be seen in Table 8, this Run subtraction degraded the AUC score, leading us to conclude that this was in fact subtracting enough signal so as to be counter–productive (see also S11 and S12 Tables). This may be due to clean air replacing the previous chemical headspace above the sample and the time taken for it to reach a new equilibrium.

**Table 8:** Model performance comparison run subtraction.

	2D DWT	Run 3—Run 1	Run 1– Run 3
AUC	**0.825**	0.606	0.605
–CIs	(0.747–0.9)	(0.498–0.71)	(0.497–0.71)
Sensitivity	0.625	0.583	0.625
–CIs	(0.264–0.497)	(0.302–0.539)	(0.264–0.497)
Specificity	0.953	0.651	0.605
–CIs	(0.00568–0.158)	(0.21–0.509)	(0.25–0.556)

Model performance (confident intervals; CIs) are reported for the Sparse Logistic Regression algorithm.

We also considered two methods for combining the information from the three Runs for each sample. The first was averaging the raw data from each Run before running the algorithm (Run ensemble). The second was to average the probabilities obtained from the classification models after each Run and then obtaining the performance metrics (Probability ensemble). The results from these two approaches are shown in Table 9, S13 and S14 Tables. As can be seen, neither of these ensembling methods produced a significant improvement in the AUC score. We therefore conclude that if there is any benefit to be gained from combining multiple runs, a more sophisticated approach than either of these simple ensembles will likely be required.

**Table 9:** Model performance comparison- noise reduction approaches.

	2D DWT	Run ensemble	Probability ensemble
AUC	**0.825**	0.808	**0.826**
–CIs	(0.747–0.9)	(0.73–0.89)	(0.752–0.9)
Sensitivity	0.625	0.708	0.653
–CIs	(0.264–0.497)	(0.19–0.411)	(0.239–0.469)
Specificity	0.953	0.814	0.907
–CIs	(0.00568–0.158)	(0.0839–0.334)	(0.0259–0.221)

Model performance (confident intervals; CIs) are reported for the Sparse Logistic Regression algorithm.

### Use of demographic data as features

We had previously investigated the performance of using the individual demographic data as predictors (see the caption in Table 1). The best performing variable among them was BMI, which achieved an AUC score of 0.78 (0.7–0.87), but it did not perform better than our baseline performance (using Run 2, *sigma* = 1*x*10^−6^, the filter method with *nKeep* = 2 and implementing a 2D DWT). We wanted to investigate whether the use of the four demographic variables alone or combined with the FAIMS features selected by the filter method improved the model performance.

Table 10, S15 and S16 Tables show the results of including the four demographic features (sex, age, alcohol use, BMI) in the analysis, both on their own and also in combination with the selected Wavelet–VOC features. The demographic data–only analysis produced a strong result (AUC = 0.87), suggesting that there is indeed confounding present in this data set due to the selection of the patient groups. However, combining demographic features and Wavelet–VOCs still leads to an improvement in AUC score (AUC = 0.897). Nevertheless, implementing a Wilcoxon rank-sum test to compare the set of prediction probabilities from both analyses showed that there is no significant difference between these for any of the classifier models (see S17 Table). Regardless, the demographic features contain relevant information for distinguishing disease from control group patients. Therefore, we recommend the inclusion of the demographic variables as part of the feature set to train the classifiers.

**Table 10:** Model performance comparison when using the demographic (demo) variables as features or when using these in addition to the two FAIMS features selected by the filter method.

	2D DWT	Demo variables	Demo and FAIMS
AUC	0.825	0.87	**0.897**
–CIs	(0.747–0.9)	(0.8–0.94)	(0.839–0.95)
Sensitivity	0.625	0.764	0.778
–CIs	(0.264–0.497)	(0.144–0.351)	(0.133–0.336)
Specificity	0.953	0.907	0.884
–CIs	(0.00568–0.158)	(0.0259–0.221)	(0.0389–0.251)

Model performance (confident intervals; CIs) are reported for the Sparse Logistic Regression algorithm.

## Conclusion

The aim of this work was to develop a data analysis pipeline to use non–invasive samples measured with a FAIMS approach to measure gases and volatile organic compounds (VOCs), leading to our recommended pipeline (Fig 2). We confirmed that a wavelet transform was an important preprocessing step and showed that using a 2D wavelet transform was notably superior to the 1D transform, which had been used in previous papers [8, 9]. We speculate that this is because the FAIMS data has a natural 2D structure, arising from the nature of the measurements.

We experimented with a 2–stage feature selection process, first screening out low variance features, and then identifying informative features via a Wilcoxon rank–sum test, a filter method. While the former was found not to be necessary, the latter is a very effective and easy to implement way of reducing the dimensionality of the data and improving AUC scores. The implementation of the filter method in the second stage resulted in better predictive classifier ability and overall reduced computation time, when compared to the implementation of an embedded or a wrapper method.

Furthermore, we investigated the effect of including principal component analysis (PCA) in the pipeline. The motivation here was to remove the effect of correlated features. However, it was found that this actually reduced the AUC scores, and we conclude that taking linear combinations of selected features may be degrading the underlying signal of interest. Additionally, we tried some simple ensembles over multiple data runs, to see if information could be usefully combined from them. These were unable to improve predictive performance, and we speculate that if any gain is to be found from such methods, a more sophisticated approach may be necessary.

Finally, we compared model performance when including the demographic variables as sole features or as additional features to those selected by the filter method. While this again showed the superior performance of the new data processing pipeline, it also identified confounding factors in the diabetes data set under consideration. This sadly limits what we can say about FAIMS ability to detect diabetes (on the basis of this data set), but we can nevertheless have some confidence that the pipeline itself is performing better than previously–used methods.

## Supporting information

S1 Table Model performance comparison using Run 1 data. Performance of the five machine learning algorithms obtained when using Run 1 data.
(PDF)

S2 Table Model performance comparison using Run 2 data. Performance of the five machine learning algorithms obtained when using Run 2 data.
(PDF)

S3 Table Model performance comparison using Run 3 data. Performance of the five machine learning algorithms obtained when using Run 3 data.
(PDF)

S4 Table Model performance comparison using FAIMS data without discrete wavelet transform. Performance of the five machine learning algorithms obtained when omitting the DWT step.
(PDF)

S5 Table Model performance comparison using FAIMS data with a 1D discrete wavelet transform. Performance of the five machine learning algorithms obtained when carrying out the 1D DWT step.
(PDF)

S6 Table Model performance comparison using FAIMS data with a 2D discrete wavelet transform (512 x 512 matrix). Performance of the five machine learning algorithms obtained when carrying out the 2D DWT step with a 512 x 512 matrix.
(PDF)

S7 Table Model performance comparison using FAIMS data with a cropped 2D discrete wavelet transform (cropped 256 x 256 matrix). Performance of the five machine learning algorithms obtained when carrying out the 2D DWT step with a 256 x 256 matrix.
(PDF)

S8 Table Model performance comparison using FAIMS data with a cropped 2D discrete wavelet transform (cropped 128 x 128 matrix). Performance of the five machine learning algorithms obtained when carrying out the 2D DWT step with a 128 x 128 matrix.
(PDF)

S9 Table Model performance comparison using FAIMS data with a 2D discrete wavelet transform and PCA. Performance of the five machine learning algorithms obtained when carrying out the 2D DWT step with a 512 x 512 matrix and PCA.
(PDF)

S10 Table Feature selection method comparison. Comparison of the performance of the five machine learning algorithms when using different feature selection methods.
(PDF)

S11 Table Sample run ensemble: Run 3—Run 1. Performance of the five machine learning algorithms obtained when carrying out run ensemble: Run 3—Run 1.
(PDF)

S12 Table Sample run ensemble: Run 1—Run 3. Performance of the five machine learning algorithms obtained when carrying out run ensemble: Run 1—Run 3.
(PDF)

S13 Table Sample run ensemble: Run Mean. Performance of the five machine learning algorithms obtained when carrying out run ensemble: Run Mean.
(PDF)

S14 Table Probability ensemble: Ensemble Mean. Performance of the five machine learning algorithms obtained when carrying out probability ensemble: Ensemble Mean.
(PDF)

S15 Table Use of demographic data as features. Performance of the five machine learning algorithms obtained when using the demographic data as features.
(PDF)

S16 Table Use of demographic data as features in addition to the 2 features selected by the filter method. Performance of the five machine learning algorithms obtained when using the demographic data as features in addition to the 2 features selected by the filter method.
(PDF)

S17 Table P–values from the Wilcoxon rank–sum test. The p–values obtained when carrying out a Wilcoxon rank–sum test for each machine learning algorithm, comparing the set of prediction probabilities obtained from the use of demographic data alone and with the two VOC features selected by the filter method.
(PDF)

S1 Fig Model performance comparison over a range of *sigma* values. Performance of the five machine learning algorithms obtained when using a range of *sigma* values on the second Run with a 2D DWT and *nKeep* value of 2. The dashed line and text value refer to the AUC achieved by the baseline parameters (Table 4 and S6 Table). It can be observed that the AUC achieved is the same or worse than the baseline, except in a few instances for the Gaussian Process and Support Vector Machine algorithms, where the AUC is fractionally higher than the baseline, but not a significant result (data not shown).
(PDF)
