# Supplementary material for: An improved machine learning pipeline for urinary volatiles disease detection: Diagnosing diabetes
Source: PLoS One. 2018 Sep 27;13(9):e0204425. doi: 10.1371/journal.pone.0204425 (PMC6160042; doi:10.1371/journal.pone.0204425)
Supplement: S1 Table — Performance of the five machine learning algorithms obtained when using Run 1 data. (PDF) [file pone.0204425.s001.pdf]

	Sparse Logistic Regression	Random Forest	Gaussian Process	Support Vector Machine	Neural Network
AUC	0.739	0.739	0.726	0.738	0.556
–CIs	(0.648 - 0.83)	(0.648 - 0.83)	(0.636 - 0.82)	(0.649 - 0.83)	(0.448 - 0.66)
Sensitivity	0.528	0.5	0.417	0.528	0.556
–CIs	(0.353 - 0.593)	(0.38 - 0.62)	(0.461 - 0.698)	(0.353 - 0.593)	(0.434 - 0.673)
Specificity	0.93	0.93	0.953	0.93	0.628
–CIs	(0.0146 - 0.191)	(0.0146 - 0.191)	(0.00568 - 0.158)	(0.0146 - 0.191)	(0.467 - 0.77)
