# Supplementary material for: An improved machine learning pipeline for urinary volatiles disease detection: Diagnosing diabetes
Source: PLoS One. 2018 Sep 27;13(9):e0204425. doi: 10.1371/journal.pone.0204425 (PMC6160042; doi:10.1371/journal.pone.0204425)
Supplement: S2 Table — Performance of the five machine learning algorithms obtained when using Run 2 data. (PDF) [file pone.0204425.s002.pdf]

	Sparse Logistic Regression	Random Forest	Gaussian Process	Support Vector Machine	Neural Network
AUC	0.825	0.783	0.757	0.804	0.803
–CIs	(0.747 - 0.9)	(0.7 - 0.87)	(0.668 - 0.85)	(0.721 - 0.89)	(0.721 - 0.88)
Sensitivity	0.625	0.5	0.667	0.639	0.611
–CIs	(0.264 - 0.497)	(0.38 - 0.62)	(0.227 - 0.454)	(0.251 - 0.483)	(0.276 - 0.511)
Specificity	0.953	0.953	0.837	0.93	0.953
–CIs	(0.00568 - 0.158)	(0.00568 - 0.158)	(0.0681 - 0.307)	(0.0146 - 0.191)	(0.00568 - 0.158)
