# Supplementary material for: An improved machine learning pipeline for urinary volatiles disease detection: Diagnosing diabetes
Source: PLoS One. 2018 Sep 27;13(9):e0204425. doi: 10.1371/journal.pone.0204425 (PMC6160042; doi:10.1371/journal.pone.0204425)
Supplement: S3 Table — Performance of the five machine learning algorithms obtained when using Run 3 data. (PDF) [file pone.0204425.s003.pdf]

	Sparse Logistic Regression	Random Forest	Gaussian Process	Support Vector Machine	Neural Network
AUC	0.805	0.751	0.703	0.793	0.738
–CIs	(0.722 - 0.89)	(0.647 - 0.85)	(0.6 - 0.81)	(0.709 - 0.88)	(0.648 - 0.83)
Sensitivity	0.833	0.792	0.708	0.847	0.514
–CIs	(0.0892 - 0.273)	(0.122 - 0.32)	(0.19 - 0.411)	(0.0788 - 0.257)	(0.367 - 0.607)
Specificity	0.674	0.744	0.674	0.628	0.884
–CIs	(0.191 - 0.485)	(0.135 - 0.412)	(0.191 - 0.485)	(0.23 - 0.533)	(0.0389 - 0.251)
