# Supplementary material for: An improved machine learning pipeline for urinary volatiles disease detection: Diagnosing diabetes
Source: PLoS One. 2018 Sep 27;13(9):e0204425. doi: 10.1371/journal.pone.0204425 (PMC6160042; doi:10.1371/journal.pone.0204425)
Supplement: S4 Table — Performance of the five machine learning algorithms obtained when omitting the DWT step. (PDF) [file pone.0204425.s004.pdf]

	Sparse Logistic Regression	Random Forest	Gaussian Process	Support Vector Machine	Neural Network
AUC	0.682	0.65	0.639	0.668	0.671
–CIs	(0.584 - 0.78)	(0.547 - 0.75)	(0.533 - 0.74)	(0.568 - 0.77)	(0.573 - 0.77)
Sensitivity	0.444	0.694	0.639	0.431	0.375
–CIs	(0.434 - 0.673)	(0.202 - 0.425)	(0.251 - 0.483)	(0.447 - 0.686)	(0.503 - 0.736)
Specificity	0.907	0.558	0.698	0.884	0.953
–CIs	(0.0259 - 0.221)	(0.291 - 0.601)	(0.172 - 0.461)	(0.0389 - 0.251)	(0.00568 - 0.158)
