# Supplementary material for: An improved machine learning pipeline for urinary volatiles disease detection: Diagnosing diabetes
Source: PLoS One. 2018 Sep 27;13(9):e0204425. doi: 10.1371/journal.pone.0204425 (PMC6160042; doi:10.1371/journal.pone.0204425)
Supplement: S5 Table — Performance of the five machine learning algorithms obtained when carrying out the 1D DWT step. (PDF) [file pone.0204425.s005.pdf]

	Sparse Logistic Regression	Random Forest	Gaussian Process	Support Vector Machine	Neural Network
AUC	0.814	0.821	0.783	0.765	0.784
–CIs	(0.736 - 0.89)	(0.744 - 0.9)	(0.7 - 0.87)	(0.678 - 0.85)	(0.701 - 0.87)
Sensitivity	0.569	0.639	0.528	0.556	0.597
–CIs	(0.314 - 0.553)	(0.251 - 0.483)	(0.353 - 0.593)	(0.327 - 0.566)	(0.289 - 0.525)
Specificity	0.977	0.907	0.953	0.93	0.907
–CIs	(0.000589 - 0.123)	(0.0259 - 0.221)	(0.00568 - 0.158)	(0.0146 - 0.191)	(0.0259 - 0.221)
