# Supplementary material for: An improved machine learning pipeline for urinary volatiles disease detection: Diagnosing diabetes
Source: PLoS One. 2018 Sep 27;13(9):e0204425. doi: 10.1371/journal.pone.0204425 (PMC6160042; doi:10.1371/journal.pone.0204425)
Supplement: S7 Table — Performance of the five machine learning algorithms obtained when carrying out the 2D DWT step with a 256 x 256 matrix. (PDF) [file pone.0204425.s007.pdf]

	Sparse Logistic Regression	Random Forest	Gaussian Process	Support Vector Machine	Neural Network
AUC	0.824	0.781	0.747	0.799	0.806
–CIs	(0.746 - 0.9)	(0.698 - 0.86)	(0.658 - 0.84)	(0.717 - 0.88)	(0.725 - 0.89)
Sensitivity	0.639	0.5	0.639	0.611	0.611
–CIs	(0.251 - 0.483)	(0.38 - 0.62)	(0.251 - 0.483)	(0.276 - 0.511)	(0.276 - 0.511)
Specificity	0.93	0.953	0.837	0.953	0.953
–CIs	(0.0146 - 0.191)	(0.00568 - 0.158)	(0.0681 - 0.307)	(0.00568 - 0.158)	(0.00568 - 0.158)
