# Supplementary material for: An improved machine learning pipeline for urinary volatiles disease detection: Diagnosing diabetes
Source: PLoS One. 2018 Sep 27;13(9):e0204425. doi: 10.1371/journal.pone.0204425 (PMC6160042; doi:10.1371/journal.pone.0204425)
Supplement: S8 Table — Performance of the five machine learning algorithms obtained when carrying out the 2D DWT step with a 128 x 128 matrix. (PDF) [file pone.0204425.s008.pdf]

	Sparse Logistic Regression	Random Forest	Gaussian Process	Support Vector Machine	Neural Network
AUC	0.824	0.784	0.742	0.801	0.777
–CIs	(0.746 - 0.9)	(0.701 - 0.87)	(0.652 - 0.83)	(0.719 - 0.88)	(0.691 - 0.86)
Sensitivity	0.597	0.5	0.625	0.597	0.556
–CIs	(0.289 - 0.525)	(0.38 - 0.62)	(0.264 - 0.497)	(0.289 - 0.525)	(0.327 - 0.566)
Specificity	0.977	0.953	0.837	0.977	0.953
–CIs	(0.000589 - 0.123)	(0.00568 - 0.158)	(0.0681 - 0.307)	(0.000589 - 0.123)	(0.00568 - 0.158)
