# Supplementary material for: An improved machine learning pipeline for urinary volatiles disease detection: Diagnosing diabetes
Source: PLoS One. 2018 Sep 27;13(9):e0204425. doi: 10.1371/journal.pone.0204425 (PMC6160042; doi:10.1371/journal.pone.0204425)
Supplement: S9 Table — Performance of the five machine learning algorithms obtained when carrying out the 2D DWT step with a 512 x 512 matrix and PCA. (PDF) [file pone.0204425.s009.pdf]

| | Sparse Logistic Regression | Random Forest | Gaussian Process | Support Vector Machine | Neural Network |
|---|---|---|---|---|---|
| AUC | 0.8 | 0.74 | 0.752 | 0.807 | 0.778 |
| –CIs | (0.717 - 0.88) | (0.65 - 0.83) | (0.663 - 0.84) | (0.728 - 0.89) | (0.693 - 0.86) |
| Sensitivity | 0.694 | 0.472 | 0.514 | 0.681 | 0.569 |
| –CIs | (0.202 - 0.425) | (0.407 - 0.647) | (0.367 - 0.607) | (0.214 - 0.44) | (0.314 - 0.553) |
| Specificity | 0.814 | 0.93 | 0.907 | 0.837 | 0.907 |
| –CIs | (0.0839 - 0.334) | (0.0146 - 0.191) | (0.0259 - 0.221) | (0.0681 - 0.307) | (0.0259 - 0.221) |
