# Supplementary material for: An improved machine learning pipeline for urinary volatiles disease detection: Diagnosing diabetes
Source: PLoS One. 2018 Sep 27;13(9):e0204425. doi: 10.1371/journal.pone.0204425 (PMC6160042; doi:10.1371/journal.pone.0204425)
Supplement: S10 Table — Comparison of the performance of the five machine learning algorithms when using different feature selection methods. (PDF) [file pone.0204425.s010.pdf]

		FILTER*	WRAPPER*						EMBEDDED**	
		2 Features	100 Features	250 Features	500 Features	1000 Features	2000 Features	3000 Features	87988 Features	
**Sparse Logistic Regression**	AUC	0.825	0.739	0.765	0.751	0.762	0.756	0.703	0.824	0.777
	–CIs	(0.747 - 0.9)	(0.645 - 0.83)	(0.672 - 0.86)	(0.652 - 0.85)	(0.672 - 0.85)	(0.666 - 0.85)	(0.603 - 0.8)	(0.746 - 0.9)	(0.694 - 0.86)
	Sensitivity	0.625	0.681	0.722	0.764	0.625	0.694	0.764	0.694	0.542
	–CIs	(0.264 - 0.497)	(0.214 - 0.44)	(0.179 - 0.396)	(0.144 - 0.351)	(0.264 - 0.497)	(0.202 - 0.425)	(0.144 - 0.351)	(0.202 - 0.425)	(0.34 - 0.58)
	Specificity	0.953	0.767	0.767	0.721	0.814	0.744	0.628	0.884	0.953
	–CIs	(0.00568 - 0.158)	(0.118 - 0.386)	(0.118 - 0.386)	(0.153 - 0.437)	(0.0839 - 0.334)	(0.135 - 0.412)	(0.23 - 0.533)	(0.0389 - 0.251)	(0.00568 - 0.158)
**Random Forest**	AUC	0.783	0.779	0.788	0.796	0.735	0.762	0.744		
	–CIs	(0.7 - 0.87)	(0.693 - 0.86)	(0.704 - 0.87)	(0.713 - 0.88)	(0.644 - 0.83)	(0.67 - 0.85)	(0.652 - 0.83)		
	Sensitivity	0.5	0.653	0.722	0.639	0.569	0.639	0.569		
	–CIs	(0.38 - 0.62)	(0.239 - 0.469)	(0.179 - 0.396)	(0.251 - 0.483)	(0.314 - 0.553)	(0.251 - 0.483)	(0.314 - 0.553)		
	Specificity	0.953	0.86	0.814	0.884	0.837	0.884	0.837		
	–CIs	(0.00568 - 0.158)	(0.053 - 0.279)	(0.0839 - 0.334)	(0.0389 - 0.251)	(0.0681 - 0.307)	(0.0389 - 0.251)	(0.0681 - 0.307)		
**Gaussian Process**	AUC	0.757	0.705	0.757	0.746	0.741	0.791	0.702		
	–CIs	(0.668 - 0.85)	(0.607 - 0.8)	(0.67 - 0.84)	(0.655 - 0.84)	(0.651 - 0.83)	(0.709 - 0.87)	(0.606 - 0.8)		
	Sensitivity	0.667	0.542	0.75	0.708	0.667	0.639	0.625		
	–CIs	(0.227 - 0.454)	(0.34 - 0.58)	(0.155 - 0.366)	(0.19 - 0.411)	(0.227 - 0.454)	(0.251 - 0.483)	(0.264 - 0.497)		
	Specificity	0.837	0.907	0.674	0.744	0.814	0.86	0.767		
	–CIs	(0.0681 - 0.307)	(0.0259 - 0.221)	(0.191 - 0.485)	(0.135 - 0.412)	(0.0839 - 0.334)	(0.053 - 0.279)	(0.118 - 0.386)		
**Support Vector Machine**	AUC	0.804	0.748	0.727	0.747	0.688	0.734	0.733		
	–CIs	(0.721 - 0.89)	(0.653 - 0.84)	(0.623 - 0.83)	(0.649 - 0.85)	(0.588 - 0.79)	(0.634 - 0.83)	(0.635 - 0.83)		
	Sensitivity	0.639	0.694	0.806	0.778	0.542	0.764	0.722		
	–CIs	(0.251 - 0.483)	(0.202 - 0.425)	(0.111 - 0.305)	(0.133 - 0.336)	(0.34 - 0.58)	(0.144 - 0.351)	(0.179 - 0.396)		
	Specificity	0.93	0.767	0.698	0.698	0.767	0.698	0.744		
	–CIs	(0.0146 - 0.191)	(0.118 - 0.386)	(0.172 - 0.461)	(0.172 - 0.461)	(0.118 - 0.386)	(0.172 - 0.461)	(0.135 - 0.412)		
**Neural Network**	AUC	0.803								
	–CIs	(0.721 - 0.88)								
	Sensitivity	0.611								
	–CIs	(0.276 - 0.511)								
	Specificity	0.953								
	–CIs	(0.00568 - 0.158)								
**Algorithm Run Time**		8.81 min	100.34 min	9.64 min	511.39 min	635.98 min	438.41 min	709.06 min	54.87 min	135.41 min

** All classifier models run*

*** Classifier models run individually*
