# Supplementary material for: An improved machine learning pipeline for urinary volatiles disease detection: Diagnosing diabetes
Source: PLoS One. 2018 Sep 27;13(9):e0204425. doi: 10.1371/journal.pone.0204425 (PMC6160042; doi:10.1371/journal.pone.0204425)
Supplement: S11 Table — Performance of the five machine learning algorithms obtained when carrying out run ensemble: Run 3—Run 1. (PDF) [file pone.0204425.s011.pdf]

	Sparse Logistic Regression	Random Forest	Gaussian Process	Support Vector Machine	Neural Network
AUC	0.606	0.626	0.622	0.615	0.677
–CIs	(0.498 - 0.71)	(0.509 - 0.74)	(0.51 - 0.73)	(0.506 - 0.72)	(0.573 - 0.78)
Sensitivity	0.583	0.833	0.597	0.639	0.75
–CIs	(0.302 - 0.539)	(0.0892 - 0.273)	(0.289 - 0.525)	(0.251 - 0.483)	(0.155 - 0.366)
Specificity	0.651	0.488	0.674	0.628	0.628
–CIs	(0.21 - 0.509)	(0.355 - 0.667)	(0.191 - 0.485)	(0.23 - 0.533)	(0.23 - 0.533)
