# Supplementary material for: An improved machine learning pipeline for urinary volatiles disease detection: Diagnosing diabetes
Source: PLoS One. 2018 Sep 27;13(9):e0204425. doi: 10.1371/journal.pone.0204425 (PMC6160042; doi:10.1371/journal.pone.0204425)
Supplement: S12 Table — Performance of the five machine learning algorithms obtained when carrying out run ensemble: Run 1—Run 3. (PDF) [file pone.0204425.s012.pdf]

	Sparse Logistic Regression	Random Forest	Gaussian Process	Support Vector Machine	Neural Network
AUC	0.605	0.629	0.623	0.617	0.624
–CIs	(0.497 - 0.71)	(0.511 - 0.75)	(0.512 - 0.74)	(0.508 - 0.73)	(0.515 - 0.73)
Sensitivity	0.625	0.889	0.569	0.653	0.681
–CIs	(0.264 - 0.497)	(0.0492 - 0.207)	(0.314 - 0.553)	(0.239 - 0.469)	(0.214 - 0.44)
Specificity	0.605	0.442	0.674	0.628	0.581
–CIs	(0.25 - 0.556)	(0.399 - 0.709)	(0.191 - 0.485)	(0.23 - 0.533)	(0.27 - 0.579)
