# Supplementary material for: An improved machine learning pipeline for urinary volatiles disease detection: Diagnosing diabetes
Source: PLoS One. 2018 Sep 27;13(9):e0204425. doi: 10.1371/journal.pone.0204425 (PMC6160042; doi:10.1371/journal.pone.0204425)
Supplement: S13 Table — Performance of the five machine learning algorithms obtained when carrying out run ensemble: Run Mean. (PDF) [file pone.0204425.s013.pdf]

	Sparse Logistic Regression	Random Forest	Gaussian Process	Support Vector Machine	Neural Network
AUC	0.808	0.741	0.733	0.798	0.804
–CIs	(0.73 - 0.89)	(0.651 - 0.83)	(0.642 - 0.82)	(0.718 - 0.88)	(0.726 - 0.88)
Sensitivity	0.708	0.528	0.667	0.667	0.653
–CIs	(0.19 - 0.411)	(0.353 - 0.593)	(0.227 - 0.454)	(0.227 - 0.454)	(0.239 - 0.469)
Specificity	0.814	0.907	0.791	0.86	0.86
–CIs	(0.0839 - 0.334)	(0.0259 - 0.221)	(0.1 - 0.36)	(0.053 - 0.279)	(0.053 - 0.279)
