# Supplementary material for: An improved machine learning pipeline for urinary volatiles disease detection: Diagnosing diabetes
Source: PLoS One. 2018 Sep 27;13(9):e0204425. doi: 10.1371/journal.pone.0204425 (PMC6160042; doi:10.1371/journal.pone.0204425)
Supplement: S14 Table — Performance of the five machine learning algorithms obtained when carrying out probability ensemble: Ensemble Mean. (PDF) [file pone.0204425.s014.pdf]

	Sparse Logistic Regression	Random Forest	Gaussian Process	Support Vector Machine	Neural Network
AUC	0.826	0.815	0.762	0.815	0.818
–CIs	(0.752 - 0.9)	(0.737 - 0.89)	(0.674 - 0.85)	(0.739 - 0.89)	(0.742 - 0.89)
Sensitivity	0.653	0.806	0.694	0.667	0.694
–CIs	(0.239 - 0.469)	(0.111 - 0.305)	(0.202 - 0.425)	(0.227 - 0.454)	(0.202 - 0.425)
Specificity	0.907	0.698	0.837	0.907	0.86
–CIs	(0.0259 - 0.221)	(0.172 - 0.461)	(0.0681 - 0.307)	(0.0259 - 0.221)	(0.053 - 0.279)
