# Supplementary material for: An improved machine learning pipeline for urinary volatiles disease detection: Diagnosing diabetes
Source: PLoS One. 2018 Sep 27;13(9):e0204425. doi: 10.1371/journal.pone.0204425 (PMC6160042; doi:10.1371/journal.pone.0204425)
Supplement: S15 Table — Performance of the five machine learning algorithms obtained when using the demographic data as features. (PDF) [file pone.0204425.s015.pdf]

	Sparse Logistic Regression	Random Forest	Gaussian Process	Support Vector Machine	Neural Network
AUC	0.87	0.896	0.848	0.87	0.619
–CIs	(0.8 - 0.94)	(0.836 - 0.96)	(0.776 - 0.92)	(0.795 - 0.94)	(0.513 - 0.73)
Sensitivity	0.764	0.847	0.736	0.833	0.972
–CIs	(0.144 - 0.351)	(0.0788 - 0.257)	(0.167 - 0.381)	(0.0892 - 0.273)	(0.00338 - 0.0968)
Specificity	0.907	0.814	0.837	0.86	0.209
–CIs	(0.0259 - 0.221)	(0.0839 - 0.334)	(0.0681 - 0.307)	(0.053 - 0.279)	(0.64 - 0.9)
