# Supplementary material for: An improved machine learning pipeline for urinary volatiles disease detection: Diagnosing diabetes
Source: PLoS One. 2018 Sep 27;13(9):e0204425. doi: 10.1371/journal.pone.0204425 (PMC6160042; doi:10.1371/journal.pone.0204425)
Supplement: S16 Table — Performance of the five machine learning algorithms obtained when using the demographic data as features in addition to the 2 features selected by the filter method. (PDF) [file pone.0204425.s016.pdf]

	Sparse Logistic Regression	Random Forest	Gaussian Process	Support Vector Machine	Neural Network
AUC	0.897	0.889	0.848	0.907	0.705
–CIs	(0.839 - 0.95)	(0.828 - 0.95)	(0.774 - 0.92)	(0.848 - 0.97)	(0.608 - 0.8)
Sensitivity	0.778	0.806	0.778	0.931	0.542
–CIs	(0.133 - 0.336)	(0.111 - 0.305)	(0.133 - 0.336)	(0.0229 - 0.155)	(0.34 - 0.58)
Specificity	0.884	0.837	0.814	0.767	0.767
–CIs	(0.0389 - 0.251)	(0.0681 - 0.307)	(0.0839 - 0.334)	(0.118 - 0.386)	(0.118 - 0.386)
