# Supplementary material for: An improved machine learning pipeline for urinary volatiles disease detection: Diagnosing diabetes
Source: PLoS One. 2018 Sep 27;13(9):e0204425. doi: 10.1371/journal.pone.0204425 (PMC6160042; doi:10.1371/journal.pone.0204425)
Supplement: S17 Table — The p–values obtained when carrying out a Wilcoxon rank–sum test for each machine learning algorithm, comparing the set of prediction probabilities obtained from the use of demographic data alone and with the two VOC features selected by the filter method. (PDF) [file pone.0204425.s017.pdf]

Sparse Logistic Regression	Random Forest	Gaussian Process	Support Vector Machine	Neural Network
0.5105	0.8274	0.9779	0.2415	0.0831
