# Supplementary material for: An improved machine learning pipeline for urinary volatiles disease detection: Diagnosing diabetes
Source: PLoS One. 2018 Sep 27;13(9):e0204425. doi: 10.1371/journal.pone.0204425 (PMC6160042; doi:10.1371/journal.pone.0204425)
Supplement: S1 Fig — Performance of the five machine learning algorithms obtained when using a range of sigma values on the second Run with a 2D DWT and nKeep value of 2. The dashed line and text value refer to the AUC achieved by the baseline parameters (Table 4 and S6 Table). It can be observed that the AUC achieved is the same or worse than the baseline, except in a few instances for the Gaussian Process and Support Vector Machine algorithms, where the AUC is fractionally higher than the baseline, but not a significant result (data not shown). (PDF) [file pone.0204425.s018.pdf]

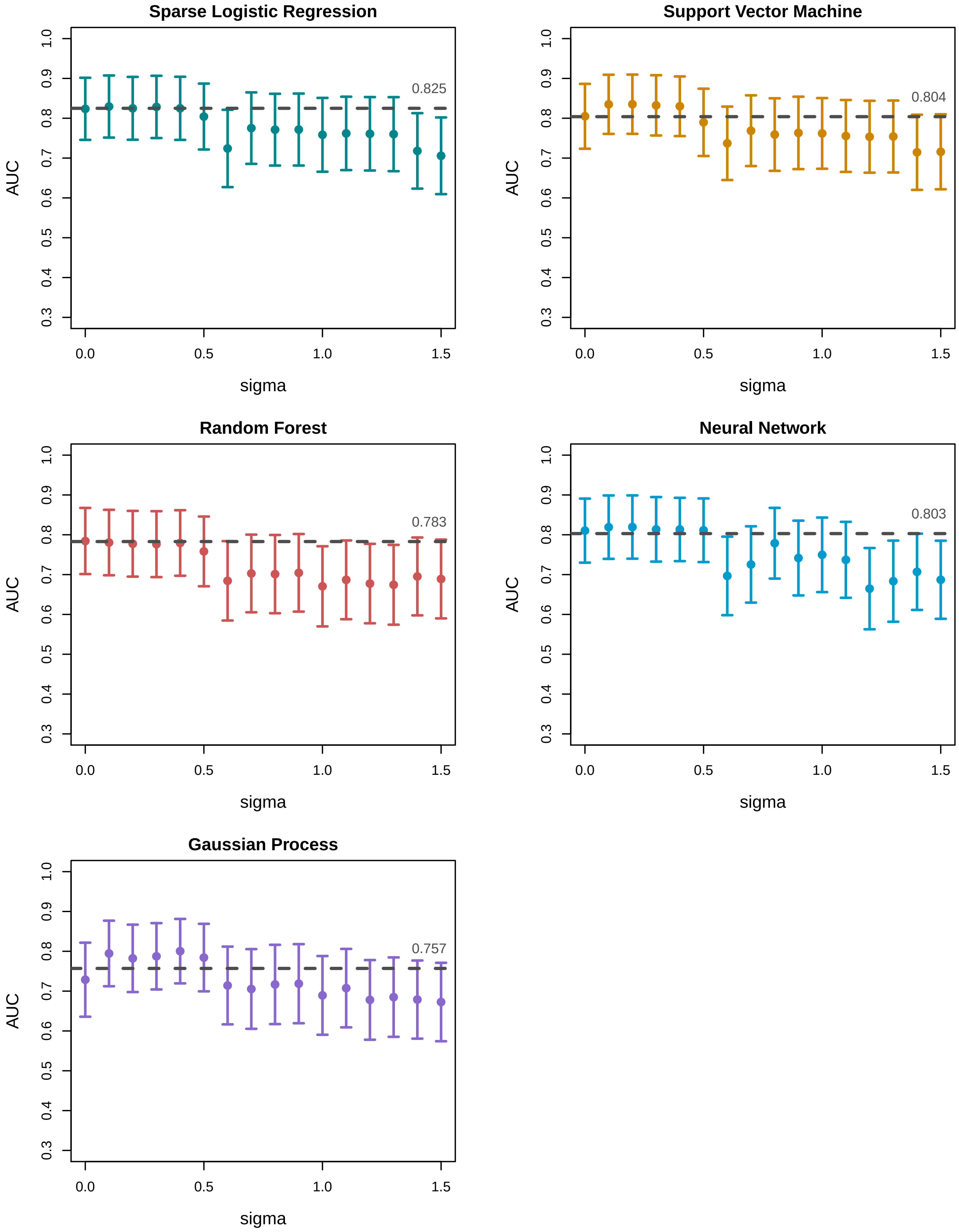
Sparse Logistic Regression
Support Vector Machine
Random Forest
Neural Network
Gaussian Process
AUC
sigma
0.825
0.804
0.783
0.803
0.757
0.3
0.4
0.5
0.6
0.7
0.8
0.9
1.0
0.0
0.5
1.0
1.5
